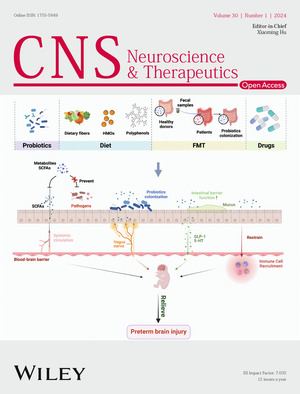# Additional Cover

**DOI:** 10.1111/cns.14625

**Published:** 2024-01-28

## Abstract

The cover image is based on the Review Article *Treatment of preterm brain injury via gut‐microbiota–metabolite–brain axis* by Ling Li et al., https://doi.org/10.1111/cns.14556.